# Age-specific differences in tumour characteristics between screen-detected and non-screen-detected breast cancers in women aged 40–74 at diagnosis in Sweden from 2008 to 2017

**DOI:** 10.1177/09691413241237616

**Published:** 2024-03-07

**Authors:** Håkan Jonsson, Anne Andersson, Zheng Mao, Lennarth Nyström

**Affiliations:** 1 Department of Radiation Sciences, Oncology, Umeå University, Umeå, Sweden; 2 Department of Epidemiology and Global Health, 8075 Umeå University, Umeå, Sweden

**Keywords:** Breast cancer, screening program, mammography, tumour characteristics, detection mode

## Abstract

**Objective:**

To analyze differences between screen-detected and non-screen-detected invasive breast cancers by tumour characteristics and age at diagnosis in the nationwide population-based mammography screening program in Sweden.

**Methods:**

Data were retrieved from the National Quality Register for Breast Cancer for 2008–2017. Logistic regression analysis was used to estimate the likelihood for a tumour to be screen-detected by tumour characteristics and age group at diagnosis.

**Results:**

In total there were 51,429 invasive breast cancers in the target age group for mammography screening of 40–74 years. Likelihood of screen detection decreased with larger tumour size, lymph node metastases, higher histological grade and distant metastasis. Odds ratios (ORs) for negative oestrogen (ER) and progesterone (PgR) were 0.41 and 0.57; for positive HER2, 0.62; for Ki-67 high versus low, 0.49. Molecular sub-types had OR of 0.56, 0.40 and 0.28, respectively, for luminal B-like, HER2-positive and triple negative versus luminal A-like. Adjusting for tumour size (T), lymph node status (N), age, year and county at diagnosis slightly elevated the ORs. Statistically significant interactions between tumour characteristics and age were found (*p* < 0.05) except for ER and PgR. The age group 40–49 deviated most from the other age groups.

**Conclusions:**

Our study demonstrates that screen-detected invasive breast cancers had more favourable tumour characteristics than non-screen-detected after adjusting for age, year and county of diagnosis, and even after adjusting for T and N. The trend towards favourable tumour characteristics was less pronounced in the 40–49 age group compared to the other age groups, except for ER and PgR.

## Introduction

Breast cancer is the most common malignancy among women worldwide. According to the National Cancer and Cause of Death Register in Sweden in 2021, 8619 women were diagnosed with breast cancer and 1326 women died with breast cancer as the underlying cause of death.^1^

Overviews and meta-analysis of the randomized controlled trials on mammography screening have shown that breast cancer screening with mammography can reduce the mortality from breast cancer for women aged 50–69 by 20–25%.^2,3^ Similar or even larger effects have been seen in evaluations of the screening service programs in Europe and New Zealand.^4–6^ Similar effects have also been found in cohort studies of the 40–49 and 70–74 year age groups in the screening program in Sweden.^7,8^ The European Commission Initiative on Breast Cancer has recently evaluated all studies on the effect of inviting women to breast cancer screening and strongly recommended biannual screening of women 50–69 years, conditionally recommended biannual/triannual screening of women 70–74 years, and conditionally recommended biannual screening of women 45–49 years.^9^

In 1986, the National Board of Health and Welfare in Sweden recommended the county councils to invite women aged 40–74 to population-based mammography screening. The recommended screening interval since 2014 is 18–24 months but earlier the recommendation was 18 months for women 40–54 and 24 months for women 55–74. The program was fully implemented in 1997 for the 50–69 age group and in 2012 for the 40–49 and 70–74 age groups.

The aim of screening is to reduce the risk of breast cancer–related deaths. However, screening programs may by early detection also affect the tumour characteristics at diagnosis, which may in turn result in less aggressive treatment.

Several studies have utilized the screening service programs with mammography in Western Europe, Canada, Australia and New Zealand to investigate differences in tumour characteristics by detection mode. These studies compared screen-detected cancers with non-screen-detected cancers, including interval cancers (IC), either independently or in conjunction with cancer cases among non-participants (NP) or those outside a screening program, often referred to as symptomatic or clinical cases. Some studies included women outside the age range 50–69 but few, if any, have studied detection mode and tumour characteristics in relation to age.

In some studies, carcinoma *in situ* was included which was more common among screen-detected cases. For invasive cancers, published studies consistently show, independent of study design and comparison group, a more favourable pattern of characteristics in screen-detected cancer, such as higher proportion of small tumour size, no regional lymph nodes, low histological grade, low Ki-67, positive oestrogen (ER) and progesterone (PgR) receptors and negative HER2 receptor.^10–23^ Among surrogate molecular sub-types the screen-detected had a higher proportion of luminal A-like cancer and lower proportion of HER2-positive and triple negative sub-type.^11,12,14,20,23^

The aim of this study was to analyze differences between screen-detected and non-screen-detected invasive breast cancers by tumour characteristics and age at diagnosis in a nationwide population-based screening program with mammography using nationwide register data.

## Material and methods

All female breast cancers, invasive as well as *in situ*, diagnosed between 2008 and 2017 were retrieved from the National Quality Register for Breast Cancer (NKBC).^24^ The NKBC is a quality register to be used in the evaluation of breast cancer care. The coverage of cancers in NKBC, that is, the proportion of cases also reported to the national cancer register (where reporting is mandatory by law) has been high, 99.3% in 2021.^25^ In the NKBC, only one tumour, regardless of invasive or *in situ*, can be recorded per breast. The register contains individual information on whether the cancer was screen-detected or non-screen-detected, tumour characteristics and age, date and residence (county) at diagnosis. Non-screen-detected include all other breast cancers than screen-detected, that is, a combination of IC and cancer among NP. The tumour characteristics used in this study were tumour size (T), lymph node status (N), distant metastases (M), histological grade, Ki-67 and receptors for ER, PgR and HER2. The status of HER2 (pos/neg) was defined by HER2 ISH (*in situ* hybridization) analysis (amplified or not). For tumours with missing data on HER2 ISH, the status was determined by immunohistochemical expression (3+ positive; 0–2 negative). Breast cancer tumours with ER >10% were defined as ER positive, otherwise ER negative. The cut-off for PgR was the same as for ER. High proliferation was set to Ki-67 > 20%, otherwise low according to earlier classification. The following breast cancer surrogate molecular sub-types were used: luminal A-like, luminal B-like, HER2-positive and triple-negative/basal-like (Table 1). Tumour characteristics were retrieved from the post-operative pathological anatomical diagnosis. For tumours lacking post-operative information regarding tumour characteristics, preoperative data were utilized as a substitute. Nonetheless, in cases of missing information for invasiveness, data on distant metastases were employed to categorize the tumour as invasive.

**Table 1.** Definition of surrogate molecular subtypes of breast cancer.

Sub-types	Tumour characteristics				
	ER status	PgR status	HER2 status	Ki-67	Histological grade
Luminal A-like	+	+	-	Low	I–II
Luminal B-like	+	+	-	Any	III
	+	+	-	High	Any
	+	-	-	Any	Any
HER2-positive	+/-	+/-	+	Any	Any
Triple-negative/basal-like	-	-	-	Any	Any

ER/PgR+: >10%; ER/PgR−: ≤10%; HER2 Pos: 3+ or amplified by ISH; HER2 Neg: 0–2+ and/or not amplified by ISH; Ki-67 Low: ≤20%; Ki-67 High: >20%.

For the age group 50–69, screening has been ongoing since the 1990s. However, for the age groups 40–49 and 70–74, screening started in some regions either just before or after the commencement of the current study. To ensure that screening had been ongoing for at least one screening round in those regions, the first two years after the start of screening were excluded.

The average mammography screening attendance rate in Sweden during 2017–2018 was 81%.^26^

### Statistical methods

Multivariable logistic regression analysis was used to estimate the odds for a tumour to be screen-detected given tumour characteristics and age group at diagnosis. Odds ratios (ORs) and 95% confidence intervals (CIs) were reported. For each tumour characteristic, the category with the best prognosis was chosen as reference category. The analysis was adjusted for calendar year, age and county at diagnosis (model I), and further also for T and N (model II).

Data were split into subgroups of age at diagnosis, 40–49, 50–59, 60–69 and 70–74, to enable adjustment for age as well as separate analyses for each age group. Interaction between age group and each tumour characteristic was tested using the likelihood ratio test. An interaction means that the ORs for a certain characteristic differed by age groups. A *p* < 0.05 was considered significant.

## Results

**Figure 1.**
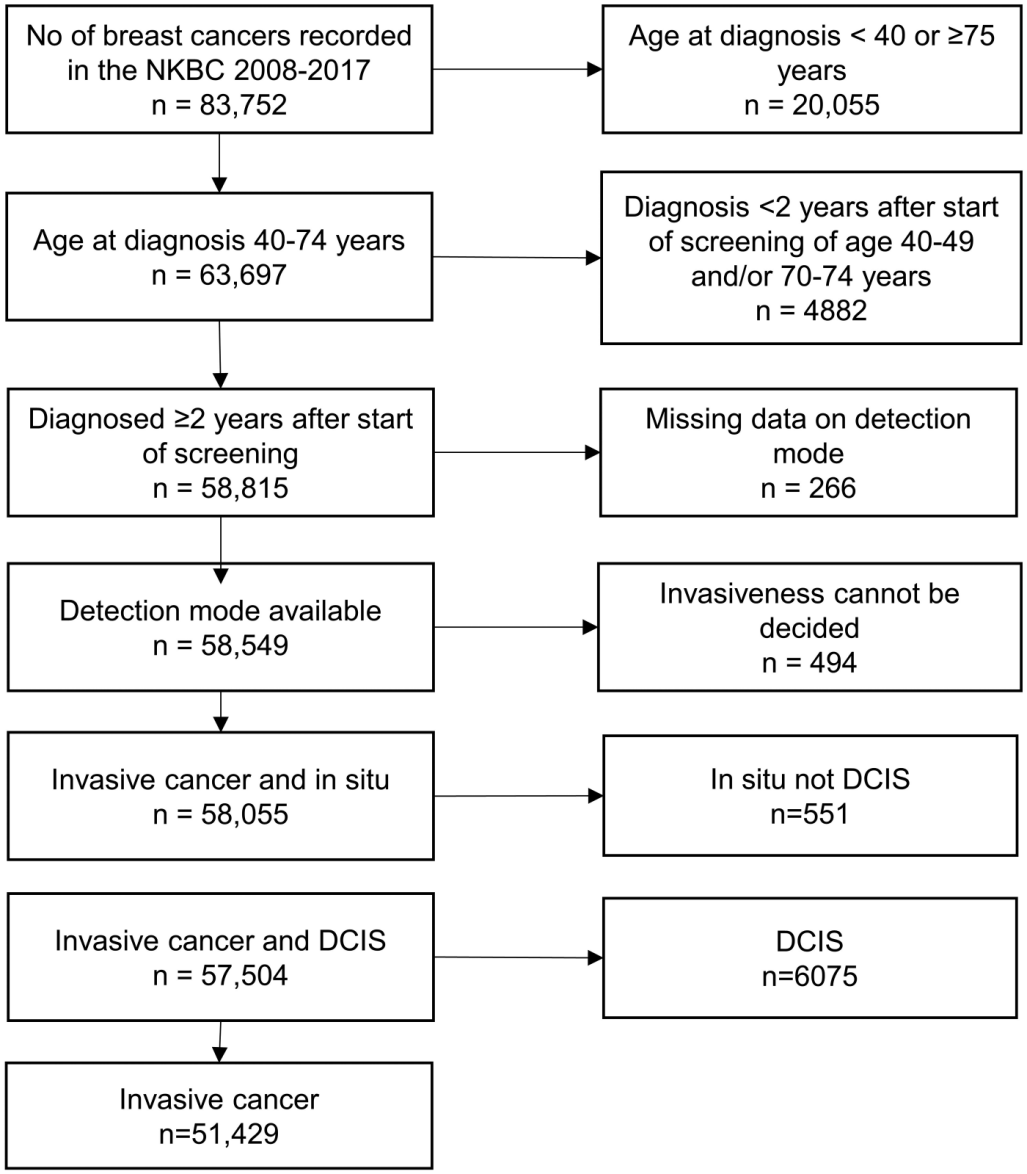
Flow chart illustrating the data generation process.

NKBC: National Quality Register for Breast Cancer; DCIS: ductal cancer in situ.

During the study period, 2008–2017, there were 83,752 breast tumours registered in the NKBC and out of these 63,697 were in women aged 40–74 at diagnosis (Figure 1). We excluded 4882 breast tumours in women aged 40–49 or 70–74 who were diagnosed earlier than two years after the regional screening program started inviting the respective age group. Further exclusion was made if the detection mode was missing (266 cases) or if information whether the tumour was invasive or not was unavailable (494 cases). Finally, 551 in-situ tumours that were not DCIS were excluded. After these exclusions, 57,504 cases remained for analysis.

In total 51,429 cases were invasive and 6075 DCIS. The number of screen-detected cases was 36,756 (64%) and 20,748 (36%) were non-screen-detected (Table 2). In the screen-detected group the percentage of DCIS decreased with increasing age from 20% in the 40–49 age group to 11% in the 70–74 age group. The corresponding figures for the non-screen-detected group were 6.6% and 5.0%. In the invasive group, the percentage of screen-detected cancers increased from 47% in the 40–49 age group to 69% in the 70–74 age group. The corresponding figures for the DCIS group were 76% and 84%.

**Table 2.** (a) Percent of DCIS in screen-detected and non-screen-detected cancer by age group. (b) Percent of screen-detected cases among invasive cancers and DCIS by age group.^a^

	Age group (years)				Total number
	40–49	50–59	60–69	70–74	
(a) Detection mode					
Screen-detected	20.0%	14.5%	11.8%	11.0%	36,756
Non-screen-detected	6.6%	5.6%	5.6%	5.0%	20,748
					
(b) Invasiveness					
Invasive	47.0%	57.6%	67.0%	69.1%	51,429
DCIS	76.0%	79.5%	81.9%	84.1%	6075
Total number	8436	15,923	24,617	9079	57,504

^a^ Additionally, the total number of cases per group is provided.

Among the invasive breast cancer cases, 1.2% lacked information on tumour size, 0.3% on lymph nodes status, 6.4% on distant metastasis status, 10% on histological grade, 2.9% on ER status, 3.1% on PgR status, 22% on HER2 and 37% on Ki-67. Due to missing data on ER, PgR, HER2 and Ki-67, surrogate molecular sub-type was not possible to determine in 40% of cases.

Number of breast cancer cases by tumour characteristics and age group at diagnosis is presented in Table 3. For all tumour characteristics, both in total and within age groups, the highest percentages of screen-detected breast cancers were observed in the category with the best prognosis (tumour size <20 mm, no regional lymph nodes, no distant metastasis, low histological grade, ER+ and PgR+, low Ki-67 and HER2−). In cases where the characteristics had more than two categories, the percentages decreased as prognosis worsened. The percentage of screen-detected cases was highest for the luminal A-like sub-type and decreased in the following order: luminal B-like, HER2-positive and triple-negative/basal-like (Table 4).

**Table 3.** Number of invasive breast cancer cases with complete data on detection mode by tumour characteristics and age group.

Tumour characteristics	Age group				Total number
	40–49	50–59	60–69	70–74	
*T-classification (size, mm)*					
T_1_: ≤-c	4462	9262	15,516	5806	35,046
T_2_: >20, ≤50	2137	3759	5225	1921	13,042
T_3_: >50	447	670	737	277	2131
T_4_: Advanced tumour	71	198	258	82	609
Total number	7117	13,889	21,736	8086	50,828
*N-classification (number of positive regional lymph nodes)*					
N_0_: 0	4596	9299	15,918	6113	35,926
N_1_: 1–3	1941	3450	4423	1487	11,301
N_2_: 4–9	462	861	1055	355	2733
N_3_: 10+	175	403	556	183	1317
Total number	7174	14,013	21,952	8138	51,277
*M-classification (distant metastasis)*					
M_0_: No	6758	12,693	19,925	7569	46,945
M_1_: Yes	135	336	521	190	1182
Total number	6893	13,029	20,446	7759	48,127
*Histological grade*					
Low	1189	2741	4838	1823	10,591
Intermediate	2983	6069	10,510	4092	23,654
High	2082	3709	4796	1594	12,181
Total number	6254	12,519	20,144	7509	46,426
*Oestrogen receptor (ER) status*					
Positive	5848	11,478	18,735	7171	43,232
Negative	1169	2141	2575	801	6686
Total number	7017	13,619	21,310	7972	49,918
*Progesterone receptor (PgR) status*					
Positive	5295	9291	14,758	5750	35,094
Negative	1711	4311	6528	2210	14,760
Total number	7006	13,602	21,286	7960	49,854
*HER2 status (ISH)*					
Neg (not amplified/0–2)	4522	9321	15,258	5119	34,220
Pos (amplified/3+)	1119	2017	2308	699	6143
Total number	5641	11,338	17,566	5818	40,363
*Ki-67*					
Low (≤20%)	2105	3903	7039	3374	16,421
High (>20%)	3217	4232	5826	2605	15,880
Total number	5322	8135	12,865	5979	32,301
*Surrogate molecular sub-type of breast cancer*					
Luminal A-like	1237	2093	3692	1616	8638
Luminal B-like	1739	3362	5573	2030	12,704
HER2-positive	1119	2017	2308	699	6143
Triple-negative/basal-like	571	1015	1334	384	3304
Total number	4666	8487	12,907	4729	30,789

**Table 4.** Percentage of screen-detected cases among invasive breast cancers by age group and tumour characteristics.^a^

Tumour characteristics	Age group					Total number
	40–49	50–59	60–69	70–74	40–74	
*T-classification (size, mm)*						
T_1_: ≤20	54	67	76	78	71	35,046
T_2_: >20, ≤50	36	42	49	51	45	13,042
T_3_: >50	32	32	34	33	33	2131
T_4_: Advanced tumour	18	12	11	15	13	609
Total number	7117	13,889	21,736	8086	50,828	50,828
*N-classification (number of positive regional lymph nodes)*						
N_0_: 0	51	64	73	74	68	35,926
N_1_: 1–3	42	49	55	58	51	11,301
N_2_: 4–9	31	39	43	50	40	2733
N_3_: 10+	28	33	40	41	37	1317
Total number	7174	14,013	21,952	8138	51,277	51,277
*M-classification (distant metastasis)*						
M_0_ : No	48	59	69	71	63	46,945
M_1_: Yes	16	10	13	16	13	1182
Total number	6893	13,029	20,446	7759	48,127	48,127
*Histological grade*						
Low	61	75	81	83	77	10,591
Intermediate	52	64	72	73	67	23,654
High	42	49	56	57	52	12,181
Total number	6254	12,519	20,144	7509	46,246	46,246
*Oestrogen receptor (ER) status*						
Positive	50	62	70	72	65	43,232
Negative	32	40	49	52	44	6686
Total number	7017	13,619	21,310	7972	49,918	49,918
*Progesterone receptor (PgR) status*						
Positive	51	63	71	73	66	35,094
Negative	34	48	59	61	53	14,760
Total number	7006	13,602	21,286	7960	49,854	49,854
*HER2 status (ISH)*						
Neg (not amplified/0–2)	48	60	69	71	64	34,220
Pos (amplified/3+)	43	50	58	59	53	6143
Total number	5641	11,338	17,566	5818	40,363	40,363
*Ki-67*						
Low (≤20%)	54	68	75	77	71	16,421
High (>20%)	42	51	61	61	55	15,880
Total number	5322	8135	12,865	5979	32,301	32,301
*Surrogate molecular sub-type of breast cancer*						
Luminal A-like	58	70	77	80	73	8638
Luminal B-like	47	57	65	66	61	12,704
HER2-positive	43	50	58	59	53	6143
Triple-negative/basal-like	30	39	50	52	43	3304
Total number	4666	8487	12,907	4729	30,789	30,789

^a^ Total (and by age group) number of invasive breast cancer cases also shown.

In univariate logistic regression analyses, the pattern consistently showed decreasing ORs with increasing tumour severity, and all ORs were statistically significant when compared to the reference category. The OR decreased with tumour size (OR: T_2_ vs. T_1 _= 0.34, T_3_ vs. T_1 _= 0.20, T_4_ vs. T_1 _= 0.06), number of lymph nodes (OR: N_1_ vs. N_0 _= 0.50, N_2_ vs. N_0 _= 0.32, N_3_ vs. N_0 _= 0.27), histological grade (intermediate vs. low = 0.60, high vs. low = 0.31) and distant metastasis (OR: M_1_ vs. M_0 _= 0.09). The OR for ER and PgR (neg vs. pos) was 0.41 and 0.57, respectively, while the OR for HER2 (pos vs. neg) was 0.62 and for Ki-67 (high vs. low) tumour it was 0.49. For molecular sub-type, the OR values were: luminal B-like vs. luminal A-like = 0.56, HER2-positive vs. luminal A-like = 0.40 and triple-negative/basal-like vs. luminal A-like = 0.28 (Table 5).

**Table 5.** Logistic regression analysis of the likelihood of invasive breast cancer being screen-detected by tumour characteristics in women aged 40–74.^a^

Characteristics	Categories	Number of		Univariate analysis		Adjusted model I		Adjusted model II	
		*SD*	NSD	OR	95%CI	OR	95%CI	OR	95%CI
T-classification	T_1_: ≤20	24,869	10,177	1		1		1	
	T_2_: >20, ≤50	5913	7129	0.34	0.33–0.35	0.34	0.33–0.36	0.39	0.37–0.41
	T_3_: >50	696	1435	0.20	0.18–0.22	0.21	0.19–0.23	0.27	0.24–0.29
	T_4_: Advanced tumour	77	532	0.06	0.05–0.08	0.06	0.04–0.07	0.08	0.06–0.10
N-classification	N_0_: 0	24,445	11,481	1		1		1	
	N_1_: 1–3	5811	5490	0.50	0.48–0.52	0.52	0.49–0.54	0.67	0.64–0.70
	N_2_: 4–9	1106	1627	0.32	0.29–0.35	0.33	0.30–0.35	0.54	0.49–0.58
	N_3_: 10+	484	833	0.27	0.24–0.31	0.27	0.24–0.30	0.52	0.46–0.59
M-classification	M_0_ : No	29,707	17,238	1		1		1	
	M_1_: Yes	155	1027	0.09	0.07–0.10	0.08	0.07–0.10	0.17	0.14–0.20
Histological grade	Low	8203	2388	1		1		1	
	Intermediate	15,919	7735	0.60	0.57–0.63	0.60	0.57–0.63	0.74	0.70–0.78
	High	6288	5893	0.31	0.29–0.33	0.32	0.30–0.34	0.45	0.42–0.48
ER status	Positive	28,298	14,934	1		1		1	
	Negative	2917	3769	0.41	0.39–0.43	0.42	0.40–0.44	0.48	0.45–0.50
PgR status	Positive	23,321	11,773	1		1		1	
	Negative	7858	6902	0.57	0.55–0.60	0.56	0.54–0.58	0.63	0.60–0.66
HER2 status	Neg (not amplified/0–2)	21,899	12,321	1		1		1	
	Pos (amplified/3+)	3227	2916	0.62	0.59–0.66	0.66	0.62–0.70	0.75	0.71–0.80
Ki-67	Low (≤20%)	11,675	4746	1		1		1	
	High (>20%)	8707	7173	0.49	0.47–0.52	0.52	0.50–0.54	0.63	0.60–0.66
Surrogate molecular sub-type	Luminal A-like	6328	2310	1		1		1	
	Luminal B-like	7714	4990	0.56	0.53–0.60	0.59	0.55–0.63	0.73	0.68–0.78
	HER2-positive	3227	2916	0.40	0.38–0.43	0.45	0.42–0.48	0.58	0.54–0.62
	Triple-negative/basal-like	1428	1876	0.28	0.26–0.30	0.30	0.28–0.33	0.37	0.34–0.41

^a^ Univariate model and multivariable model adjusted for age, year and county at diagnosis (model I) and age, year, county at diagnosis and T and N (model II). Number of screen-detected (SD) and non-screen detected (NSD) cases, odds ratio (OR) and 95% confidence intervals (CIs).

Adjusting the OR for age, year and county at diagnosis (model I) did not change the pattern. Further adjustment for T and N (model II) slightly elevated the ORs, and thus the differences became somewhat reduced but still statistically significant (Table 5).

The OR increased significantly by age (OR: 50–59 vs. 40–49 = 1.5, 95% CI (1.5–1.6); 60–69 vs. 40–49 = 2.3, 95% CI (2.2–2.4); 70–74 vs. 40–49 = 2.5, 95% CI (2.4–2.7)).

The analysis was also conducted individually for each of the age groups 40–49, 50–59, 60–69 and 70–74 years (Table 6). The age-specific ORs were statistically significant and decreased as tumour severity increased. The interactions between each tumour characteristic and age were tested. All interactions were statistically significant except for ER and PgR. All significant tumour characteristics showed a decreasing age-specific OR with increasing age. However, there was an observed larger disparity in age-specific ORs between the age groups 40–49 and 50–59 than between the age groups 50–59, 60–69 and 70–74.

**Table 6.** Logistic regression analysis of the likelihood of invasive breast cancer being screen-detected by tumour characteristics in women aged 40–49, 50–59, 60–69 and 70–74 at diagnosis.^a^

Tumour characteristics	Categories	40–49		50–59		60–69		70–74		*p* value
		OR	CI	OR	CI	OR	CI	OR	CI	
T-classification	T_1_: ≤20	1		1		1		1		<0.0001
	T_2_: >20, ≤50	0.48	0.44–0.54	0.36	0.34–0.39	0.31	0.29–0.33	0.29	0.26–0.33	
	T_3_: >50	0.39	0.32–0.48	0.23	0.20–0.28	0.16	0.14–0.19	0.14	0.11–0.18	
	T_4_: Advanced tumour	0.19	0.10–0.35	0.07	0.04–0.10	0.04	0.03–0.06	0.05	0.02–0.09	
N-classification	N_0_: 0	1		1		1		1		<0.0001
	N_1_: 1–3	0.68	0.61–0.76	0.53	0.49–0.58	0.46	0.43–0.50	0.48	0.43–0.54	
	N_2_: 4–9	0.43	0.35–0.53	0.36	0.31–0.41	0.28	0.24–0.31	0.34	0.28–0.43	
	N_3_: 10+	0.37	0.26–0.51	0.28	0.23–0.35	0.25	0.21–0.30	0.24	0.18–0.33	
M-classification	M_0_ : No	1		1		1		1		0.0042
	M_1_: Yes	0.20	0.13–0.32	0.08	0.06–0.11	0.07	0.05–0.09	0.08	0.05–0.12	
Histological grade	Low	1		1		1		1		<0.0001
	Intermediate	0.67	0.59–0.77	0.59	0.53–0.65	0.60	0.55–0.65	0.57	0.49–0.65	
	High	0.46	0.40–0.53	0.32	0.29–0.36	0.30	0.28–0.33	0.27	0.23–0.32	
ER status	Positive	1		1		1		1		0.44
	Negative	0.46	0.41–0.53	0.42	0.38–0.46	0.41	0.38–0.44	0.43	0.37–0.50	
PgR status	Positive	1		1		1		1		0.13
	Negative	0.50	0.45–0.56	0.54	0.50–0.58	0.58	0.54–0.62	0.56	0.51–0.62	
HER2 status	Neg (not amplified/0–2)	1		1		1		1		0.0050
	Pos (amplified/3+)	0.81	0.71–0.92	0.66	0.60–0.73	0.62	0.57–0.68	0.58	0.50–0.69	
Ki-67	Low (≤20%)	1		1		1		1		0.0028
	High (>20%)	0.63	0.56–0.70	0.50	0.46–0.55	0.52	0.48–0.56	0.48	0.42–0.53	
Surrogate molecular sub-type	Luminal A-like	1		1		1		1		0.019
	Luminal B-like	0.66	0.57–0.76	0.56	0.50–0.63	0.56	0.51–0.62	0.47	0.40–0.54	
	HER2-positive	0.55	0.47–0.65	0.42	0.37–0.48	0.40	0.36–0.45	0.34	0.28–0.42	
	Triple-negative /basal-like	0.32	0.26–0.39	0.27	0.23–0.31	0.29	0.26–0.33	0.27	0.21–0.34	

^a^ Odds ratio (OR) and 95% confidence intervals (CIs). *P*-values for likelihood ratio test of interaction between age and each tumour characteristic.

Adjusting for the prognostic factors T and N in the model including interaction between tumour characteristics and age group resulted in ORs somewhat closer to one. Furthermore, there were no longer significant interactions with age for distant metastasis, Ki-67 and molecular sub-types. The larger disparities in age-specific ORs between the age group 40–49 and the other age groups remained in T, N, histological grade and HER2 (Table 7).

**Table 7.** Logistic regression[110] analysis of the likelihood of invasive breast cancer being screen-detected by tumour characteristics in women aged 40–49, 50–59, 60–69 and 70–74 at diagnosis adjusted for year, county, T and N.^a^

Tumour characteristics	Categories	40–49		50–59		60–69		70–74		*p* value
		OR	CI	OR	CI	OR	CI	OR	CI	
T-classification	T_1_: ≤20	1		1		1		1		<0.0001
	T_2_: >20, ≤50	0.54	0.48–0.60	0.41	0.38–0.44	0.35	0.33–0.38	0.33	0.30–0.37	
	T_3_: >50	0.49	0.40–0.61	0.30	0.25–0.35	0.21	0.18–0.24	0.18	0.14–0.24	
	T_4_: Advanced tumour	0.21	0.11–0.40	0.08	0.05–0.13	0.06	0.04–0.09	0.07	0.04–0.13	
N-classification	N_0_: 0	1		1		1		1		<0.0001
	N_1_: 1–3	0.90	0.81–1.01	0.67	0.62–0.73	0.60	0.56–0.65	0.64	0.57–0.73	
	N_2_: 4–9	0.66	0.53–0.82	0.57	0.49–0.67	0.46	0.40–0.52	0.58	0.46–0.73	
	N_3_: 10+	0.69	0.49–0.98	0.52	0.42–0.65	0.48	0.40–0.58	0.49	0.36–0.68	
M-classification	M_0_: No	1		1		1		1		0.056
	M_1_: Yes	0.34	0.20–0.57	0.15	0.10–0.22	0.14	0.11–0.19	0.18	0.12–0.28	
Histological grade	Low	1		1		1		1		0.0004
	Intermedia	0.84	0.73–0.96	0.73	0.66–0.81	0.73	0.67–0.80	0.70	0.60–0.80	
	High	0.62	0.53–0.72	0.44	0.40–0.50	0.42	0.38–0.46	0.39	0.34–0.46	
ER status	Positive	1		1		1		1		0.86
	Negative	0.50	0.43–0.57	0.47	0.43–0.52	0.47	0.43–0.51	0.49	0.42–0.58	
PgR status	Positive	1		1		1		1		0.076
	Negative	0.56	0.50–0.63	0.61	0.56–0.66	0.66	0.62–0.71	0.64	0.57–0.71	
HER2 status	Neg (not amplified/0–2)	1		1		1		1		0.021
	Pos (amplified/3+)	0.92	0.80–1.06	0.73	0.66–0.81	0.72	0.65–0.79	0.71	0.60–0.85	
Ki-67	Low (≤20%)	1		1		1		1		0.051
	High (>20%)	0.73	0.65–0.81	0.60	0.55–0.66	0.62	0.57–0.67	0.59	0.53–0.67	
Surrogate molecular sub-type	Luminal A-like	1		1		1		1		0.096
	Luminal B-like	0.82	0.70–0.95	0.75	0.66–0.84	0.73	0.66–0.81	0.60	0.51–0.71	
	HER2-positive	0.71	0.60–0.85	0.57	0.50–0.66	0.56	0.50–0.63	0.49	0.40–0.60	
	Triple-negative /basal-like	0.39	0.31–0.48	0.36	0.30–0.42	0.39	0.34–0.45	0.35	0.28–0.45	

^a^ Odds ratio (OR) and 95% confidence intervals (CI). *P*-values for likelihood ratio test of interaction between age and each tumour characteristic.

## Discussion

The relationship between tumour characteristics and whether the breast cancer was screen-detected or not was studied among women diagnosed at age 40–74 in the Swedish population-based mammography screening programme.

The proportion of screen-detected breast cancer increased with age and was higher among DCIS cases than invasive cancers. There was a higher proportion of small tumour size, no lymph node metastasis, no distant metastasis, low histological grade, low Ki-67, positive ER and PgR and negative HER2 in screen-detected invasive cancers. The proportion screen-detected was highest for the molecular sub-type luminal A-like and lowest in triple-negative/basal-like cancer. Adjusting for T and N factors slightly diminished the disparities in the likelihood of screen detection for invasive cancer.

The age-specific ORs varied significantly by age group, with the highest ORs observed in the 40–49 group, except for ER and PgR which were not statistically significant. After adjustment for T and N, the interactions with age for T (adjusted for N), N (adjusted for T), histological grade and HER2 remained statistically significant.

Although our study, with over 51,000 invasive breast cancers, was larger than most studies (only that by Farshid and Walters^20^ was larger), smaller studies show consistent and most often statistically significant differences for all characteristics including age, similar to our results. Bucchi et al. compared results for the risk of axillary lymph node metastases for screen-detected cases versus clinical cases with and without adjustment for tumour size.^18^ Similar to our result, the OR became closer to one but still statistically significant after adjustment. We are not aware of any other study having compared age-specific results for the OR of screen-detected versus non-screen-detected cases for tumour characteristics.

The proportion of screen-detected cases was lowest in women aged 40–49 at diagnosis. One explanation might be a higher proportion of women with dense breasts in this age group leading to lower sensitivity of the screening program. However, dense breasts cannot explain the higher OR in young women observed for many of the tumour characteristics. Despite the large number of cases in the study, no statistically significant differences in age-specific ORs were found for ER or PgR status.

Cancer cases in the non-screen-detected group constituted a mix of IC and NP in screening. The proportions of these two groups were unknown; however, we utilized the number of cases of these two groups diagnosed at age 50–69 in Stockholm 1989–2014 to estimate the proportion of non-screen-detected IC in the current study.^27^ Assuming the number of IC and cancers among NP to be proportional to the number of attenders and NP, respectively, in the population the proportion of IC is (IC*p/0.73)/(IC*p/0.73+NP*(1-*p*)/0.27), where IC = 3538 and NP = 2805 in the Stockholm data, where the participation rate was 0.73, while *p* is the actual participation rate. Given a participation rate of 81% in the current study the estimated percentage of IC in the non-screen detected group was 67%. Thus, 88% (0.64 + 0.67*0.36 = 0.88) of breast cancers in the target age group were related to participation to screening. One possible explanation for the difference (81% vs. 88%) is that as long as screening is ongoing, where future cases are detected earlier, the incidence would be higher as long as periods where screening has stopped, for example, after age 74, are not included. Another explanation is that the decision to participate or not is due to self-selection, which may lead to a different breast cancer incidence among NP compared to participants. A third explanation may be overdiagnosis.

### Strengths

The mammography screening program in Sweden is population-based and women aged 40–74 are personally invited by a letter every 18–24 months. The data source was the NKBC which has a very high coverage of breast cancer cases when compared to the national cancer register where reporting is mandatory by law.^18^ The study is large and thus has the statistical power to analyze the proportion of screen-detected in comparison with non-screen-detected invasive breast cancer by different tumour characteristics with and without interaction with age. Potential confounders including T and N were adjusted for in the model.

### Weakness

Data in NKBC have been validated by the six Regional Cancer Centres. In total, 800 breast cancer cases diagnosed in 2013 were randomly selected. A 5% disagreement in mode of detection (screen-detected/non-screen-detected) was found.^28^ The misclassification might bias the differences in the proportion of screen-detected and non-screen-detected cases, but it is probably not related to tumour characteristics, that is, no differential misclassification.

Opportunistic screening exists in Stockholm County, but maybe also in Gothenburg and Malmö. Its magnitude is however unknown. It may cause a lower rate of registered screen-detected cases which might lead to an underestimation of the difference between screen-detected and non-screen detected cases.

The relatively high proportion of missing data for HER2 and Ki-67, which also resulted in missing information on molecular sub-type, appears to be largely related to time. For HER2, the proportion of missing data was less than 10% in 2008–2014 but in following years increased to almost 50%. Conversely, for Ki-67, there was a high proportion of missing data in 2008–2012, but from 2013 onwards it was around 2%. Thus, the main source of missing data is attributed to changes in registration policies over time and is not directly related to the nature of cancer. For the remaining missing data, there may be a selection bias due to tumour characteristics, but it is likely not associated with whether the cancer was detected by screening or not. Therefore, differential misclassification appears to be a minor concern.

### Adjustment for T and N

Large, non-symptomatic tumours are often easier to detect on a mammogram compared to smaller ones. Furthermore, tumours having visible axillary lymph node metastasis on the mammogram, with or without observed abnormality in the breasts, will probably be selected for further examination. Thus, the relationship between screen detection and tumour characteristics may be influenced by both T and N.

### Causality

The results consistently show that tumours with a better prognosis more often are screen-detected. There may be two explanations for this. One possibility is that as tumours grow, their characteristics shift towards a less favourable pattern. The other explanation may be that tumours with favourable characteristics grow more slowly, increasing the likelihood of being detected through screening compared to faster-growing tumours, so-called length bias. Nevertheless, even after adjustments for T and N, the association between screen detection and tumour characteristics persisted, although it was somewhat weakened. Thus, some, but not all, of the differences in screen detection observed for the tumour characteristics could be explained by T and N.

### Screening 40–41 years

In the age range 40–49, there might have been situations where individuals aged 40–41 received a breast cancer diagnosis before their initial screening invitation. As a result, the age group 40–41 shows certain distinctions from the rest of the cases in this age range. We examined the outcomes by excluding the age 40–41 group from the 40–49 age range, and the disparities from the aforementioned results are generally minor. The main differences are that the result for HER2 in Table 6 was not significant (*p* = 0.059), and in Table 7, HER2 was not significant (*p* = 0.18) while PgR was significant (*p* = 0.044).

## Conclusion

In conclusion, the present study has illustrated that specific tumour characteristics of invasive breast cancer are associated with a higher proportion of screen-detected cases including small tumour size, absence of lymph node metastasis, lack of distant metastasis, low histological grade, low Ki-67 expression and positive receptor status for ER and PgR, and negative status for HER2. The highest proportion of screen-detected cases was observed in the luminal A-like sub-type, while the lowest was in the triple-negative/basal-like sub-type. Furthermore, the variations in the proportion of screen-detected cancers based on tumour characteristics also exhibited significant divergence across different age groups, except in the case of ER and PgR status. The trend towards more favourable tumour characteristics was less pronounced in the age group 40–49 compared to the other age groups.
